# Promotion of Adrenal Pheochromocytoma (PC-12) Cell Proliferation and Outgrowth Using Schwann Cell-Laden Gelatin Methacrylate Substrate

**DOI:** 10.3390/gels8020084

**Published:** 2022-01-28

**Authors:** Yuye Huang, Kailei Xu, Jingyi Liu, Guangli Dai, Jun Yin, Peng Wei

**Affiliations:** 1 Department of Plastic and Reconstructive Surgery, Ningbo First Hospital, Ningbo 315010, China; huangyy63@mail2.sysu.edu.cn (Y.H.); xukailei@knights.ucf.edu (K.X.); 2 Center for Medical and Engineering Innovation, Central Laboratory, Ningbo First Hospital, Ningbo 315010, China; 3 The State Key Laboratory of Fluid Power and Mechatronic Systems, School of Mechanical Engineering, Zhejiang University, Hangzhou 310028, China; 11725024@zju.edu.cn; 4 Key Laboratory of 3D Printing Process and Equipment of Zhejiang Province, School of Mechanical Engineering, Zhejiang University, Hangzhou 310028, China; 5 Department of Medical Engineering, Ningbo First Hospital, Ningbo 315010, China; daiguangli@126.com

**Keywords:** gelatin methacrylate, 3D culture, Schwann cells, neurotrophic factors, nerve regeneration

## Abstract

Peripheral nerve injuries cause different degrees of nerve palsy and function loss. Due to the limitations of autografts, nerve tissue engineering (TE) scaffolds incorporated with various neurotrophic factors and cells have been investigated to promote nerve regeneration. However, the molecular mechanism is still poorly understood. In this study, we co-cultured Schwann cells (SCs) and rat adrenal pheochromocytoma (PC-12) cells on 50% degrees of methacryloyl substitution gelatin methacrylate (GelMA) scaffold. The SCs were encapsulated within the GelMA, and PC-12 cells were on the surface. A 5% GelMA was used as the co-culture scaffold since it better supports SCs proliferation, viability, and myelination and promotes higher neurotrophic factors secretion than 10% GelMA. In the co-culture, PC-12 cells demonstrated a higher cell proliferation rate and axonal extension than culturing without SCs, indicating that the secretion of neurotrophic factors from SCs can stimulate PC-12 growth and axonal outgrowth. The mRNA level for neurotrophic factors of SCs in 5% GelMA was further evaluated. We found significant upregulation when compared with a 2D culture, which suggested that this co-culture system could be a potential scaffold to investigate the mechanism of how SCs affect neuronal behaviors.

## 1. Introduction

Millions of people worldwide are injured in traffic, sports, and military accidents every year, which commonly leads to peripheral nerve injuries (PNIs). PNIs may result in disabilities, negatively impacting sufferers’ quality of life [1,2]. Peripheral nerves can generally self-heal from mild and moderate traumas. However, it generally becomes more complicated when the trauma is greater than 5 mm [3,4]. Autografts is the gold standard for the clinical treatment of PNIs for the past decades due to the low immunogenicity, promotion of cell adhesion and migration, vascularization, and axonal extension. However, they still suffer limitations, including nerve unavailability, size mismatch, and local tissue adhesion [5,6,7,8], which significantly affect nerve rehabilitation outcomes.

The development of tissue engineering (TE) has led to a novel direction for PNI treatment. Tissue engineering is to construct artificial biological tissues in vitro by isolating suitable cells from donor tissues and combining with biocompatible scaffold materials. Hydrogel [9,10] and aerogels [11,12] are both commonly used biomaterials in TE, which have customized physical and biochemical properties that mimic natural extracellular niches or high flexibility and porosity. For example, artificial nerve conduits can connect the two ends of the injured nerves to support and promote nerve regeneration [13,14,15]. In nerve TE, neurotrophic factors and cells in the nerve microenvironment play critical roles in nerve regeneration [16]. Neurotrophic factors, including nerve growth factor (NGF), brain-derived neurotrophic factor (BDNF), glial cell line-derived neurotrophic factor (GDNF), and neurotrophin 3 (NT3), have shown their ability to promote nerve regeneration. NGF secretion in target organs of sensory and sympathetic nerves promotes sensory ganglia and nerve cell survival [17,18]. BDNF expression is upregulated in PNIs, promoting the survival and outgrowth of sensory, sympathetic, and motor nerves [19]. Other neurotrophic factors, i.e., ciliary neurotrophic factor (CNTF), insulin-like growth factors, and fibroblast growth factors, have been shown to promote nerve regeneration as well [20,21]. In a nerve TE experiment, neurons were cultured on TE scaffolds infiltrated with neurotrophic factors. Specifically, NGF was incorporated with gelatin [22,23], chitosan [24], and collagen [25] for nerve cell 3D culture and demonstrated a positive effect on cellular proliferation and axonal extension. CNTF and NGF-conjugated on silk electrospun scaffolds were also developed for the investigation of their bifunctional effects on cellular behaviors [26].

Schwann cells (SCs) cultured on nerve TE scaffolds expressed higher neurotrophic factor gene expression and protein secretion. Since SCs are glial cells of the peripheral nervous system (PNS), SCs transplantation has shown therapeutic potential in promoting axon regeneration and myelination after peripheral nerve and central nervous system injury [27,28]. SCs cultured in gelatin-alginate hydrogels have demonstrated higher mRNA levels of BDNF, GDNF, and platelet-derived growth factor (PDGF) and more secretion of NGF when compared with 2D cultures [29]. The co-transplantation of SCs and neurons in the injured spinal cord also facilitates neuron survival and axonal regeneration [30,31]. Although the functions of neurotrophic factors in nerve regeneration and 3D cell culture of neurotrophic cells have been investigated, the co-culture model of SCs and neuron cells has been rarely developed, and the molecular mechanism of how SCs affect neuron cell behaviors need to be extensive illustrated. Three-dimensional cell culture could better simulate the in vivo microenvironment which may benefit for cell proliferation, differentiation, cell morphology, and gene expression compared with 2D culture; furthermore, it also provides a better in vitro model for clinical drug screening [32].

In this study, we co-cultured SCs and rat pheochromocytoma (PC-12) cells on a gelatin methacrylate (GelMA) scaffold to determine SCs secretions’ effect on PC-12 cells. We embedded SCs in GelMA while concomitantly culturing the PC-12 cells on the GelMA surface. GelMA is a widely used biomaterial in TE due to its good biocompatibility, biodegradability, and 3D printability [33,34]. In addition, neurons have been cultured on different stiffnesses of GelMA to explore the effect of substrate stiffness on neuronal outgrowth [35]. In previous reports, the additive-lathe 3D-bioprinted GelMA embedded with bone marrow stem cells and nerve cells has shown great potential in facilitating peripheral nerve repair [15]. For biological analysis, SCs proliferation rate was analyzed with Cell Counting Kit-8 (CCK-8); the secretion of neurotrophic growth factors was evaluated by ELISA assay; and the mRNA level of neurotrophic growth factors was analyzed by qPCR. In addition, the morphology of PC-12 cells was determined by immunofluorescent staining, and CCK-8 analyzed the proliferation rate. These models provided a promising method for a highly reproducible cell encapsulation system with supportive cells for peripheral nerve regeneration.

## 2. Result and Discussion

### 2.1. Material Characterization of GelMA

**Figure 1:**
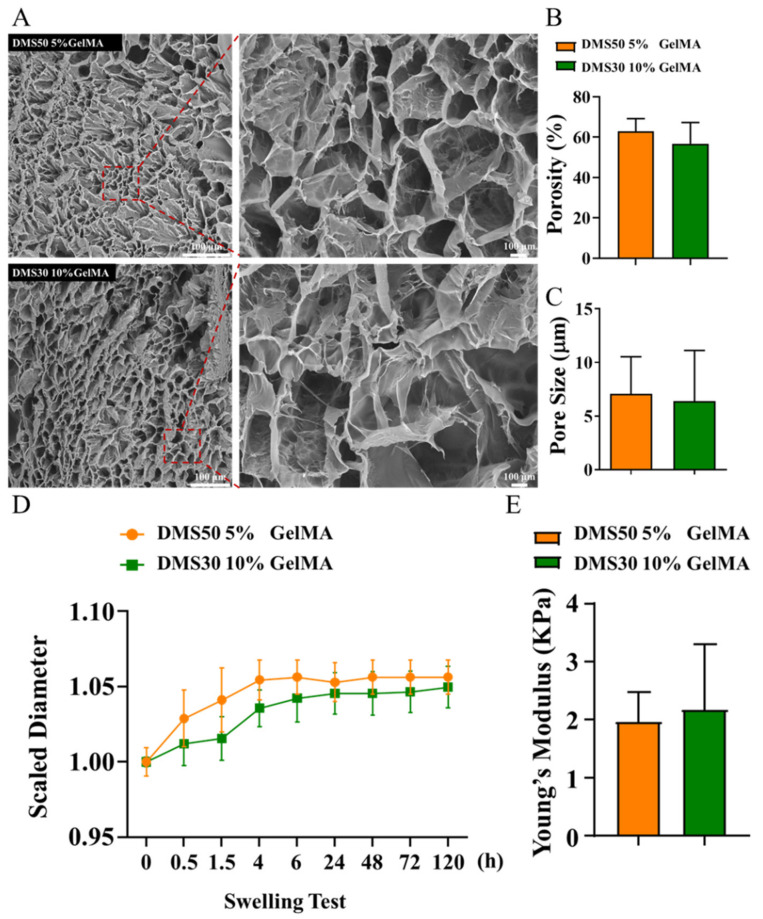
Material characterization. (**A**) The surface strucures GelMA, (**B**) Pore size of GelMA, (**C**) Porosity of GelMA, (**D**) Swelling test for GelMA, (**E**) Young’s modulus for GelMA under compression test.

The GelMA stiffness, hardness, porosity, and pore size could be adjusted with the appropriate DMS (20–80%) and concentration [36,37]. To investigate the suitbale GelMA compositions for SCs cell growth, SCs were cultured in 5% GelMA (50% DMS) and 10% GelMA (30% DMS) for material selection before developing the co-culture system. The surface structures of 5% and 10% GelMA were characterized with SEM under 250× and 1000× (Figure 1A). Both GelMA compositions showed honeycomb-like structures with highly interconnected porous networks, which enable nutrient diffusion and facilitates cell proliferation. The transportation of biological molecules through hydrogels almost solely depends on diffusion of solutes. The porosities of GelMA hydrogels are 62.85% ± 6.30% and 56.74% ± 10.57% (Figure 1B) for 5% and 10% GelMA, respectively, which were corresponding to previous studies [38]. The average pore size was 7.1 ± 3.5 μm, and 6.4 ± 4.7 μm for 5% and 10% GelMA (Figure 1C), respectively, which was similar to other research [39,40]. It is worthy of noting that the 5% GelMA hydrogel had larger pore sizes than 10% GelMA, which could better support the cellular growth.

The swelling properties of hydrogels are indicative of the diffusivity of fluids through the hydrogels [38]. According to Figure 1D, the scaled diameter of both GelMA dicks increase with time after being soaked in fully supplemented media, which indicates that both GelMA had good swelling properties. Furthermore, the swelling ratios of two GelMA compositions were equivalent, which was consistent with the pore structures observation. For the mechanical characterization of 5% and 10% GelMA, unconfined compression tests are conducted using a dynamic mechanical analysis instrument (ElectroForce, TA Instruments, New Castle, DE, USA) at room temperature. The compressive modulus was calculated as the slope of the linear region in the 0–10% strain range of the stress-strain curves. For the two GelMA hydrogels, it can be found that the compressive modulus is very small (<2.5 KPa), typical of soft materials (Figure 1E).

### 2.2. Encapsulation of SCs in GelMA

GelMA is a commonly used hydrogel with tunable and cost-efficient advantages, and possesses an endogenous extracellular matrix (ECM) [41,42]. Compared with other biomaterials used for nerve TE, such as alginate [43] and PLLA (Poly-l-lactic acid) [44], GelMA retains the bioactive sequences of collagen that could better promote the adhesion, proliferation, and migration of nerve cells [45,46,47]. SCs were found to have enhanced myelination on GelMA hydrogel even compared with collagen [48], indicating that GelMA could be a promising biomaterial for developing the nerve TE scaffold. Since the biological properties of GelMA were tunable through the modification of DMS and related reagents, SCs were cultured in 5% GelMA (50% DMS) and 10% GelMA (30% DMS) for material selection.

**Figure 2:**
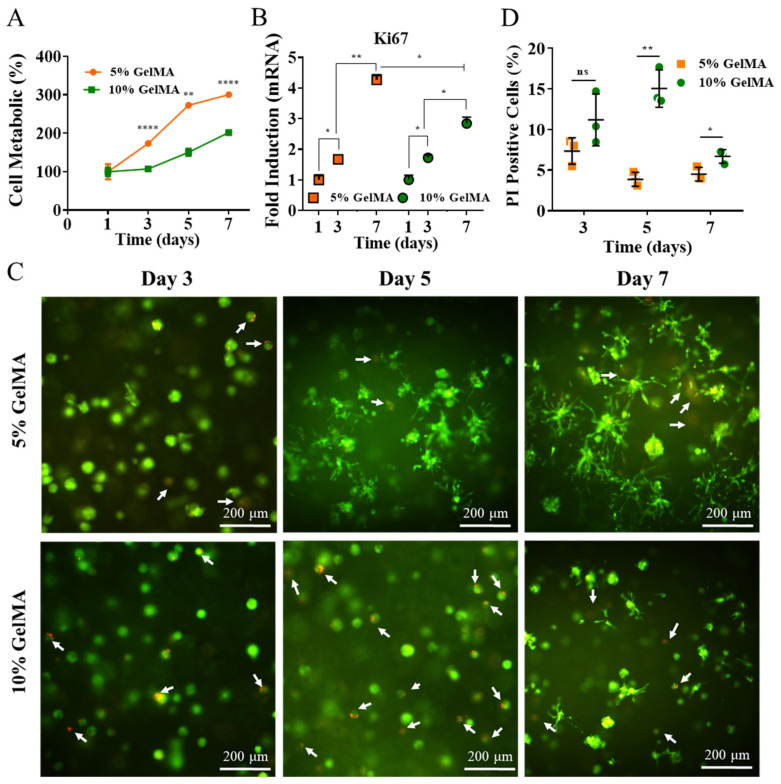
SCs proliferation and viability in 5% and 10% GelMA. (**A**) CCK-8 result of SCs in 5% and 10% GelMA on day 1, 3, 5, and 7. (**B**) qPCR result of ki67 expression of SCs in 5% and 10% GelMA on day 1, 3, and 7. (**C**) Statistical cell viability. Quantification of PI-positive cells (dead cells). (**D**) Live/Dead assay by calcein-AM and PI staining on day 3, 5, and 7. Living cells are depicted in green, and dead cells are in red. (Scale bar: 200 μm). * *p* < 0.05, ** *p* < 0.01, **** *p* < 0.0001.

The cellular proliferation was determined with CCk-8 assay at days 3, 5, and 7, which suggested that 5% and 10% GelMA can successfully supported cellular growth. The cellular proliferation rates of SCs embedded in 5% GelMA were 1.7-, 2.7-, and 3-fold changes for days 3, 5, and 7, respectively, normalized to day 1 (Figure 2A). The SCs grew significantly from day 3 to day 5 and became slower from day 5 to day 7. On the other hand, the cellular proliferation rates of SCs embedded in 10% GelMA demonstrated steady growth, with 1.05-, 1.5-, and 2-fold changes for days 3, 5, and 7, respectively, normalized to day 1 (Figure 2A). The mRNA level of the proliferation-related gene, ki67 of SCs, was also analyzed by real-time PCR to identify further SCs proliferation in 5% and 10% GelMA. Figure 2B showed that the ki67 gene expression had the same tendency as the CCK-8 assay. However, fold changes of ki67 mRNA level in SCs in 5% GelMA increased faster than that in 10% GelMA from day 3 to day 7, with the relative mRNA of ki67 expression reaching fourfold on day 7, while the fold change for 10% GelMA was only three. Overall, SCs demonstrated a significantly higher cell proliferation preference in 5% GelMA than in 10% GelMA at all time points. These results may be caused by denser crosslink networks present in the 10% GelMA, as denser crosslinks may mechanically inhibit cell growth and nutrient transport [47]. This was corresponding to the results found in SCs encapsulated in alginate-based hydrogel [43]. Similar results were also reported in our previous study [15].

Furthermore, we used a Live/Dead viability assay on days 3, 5, and 7 to investigate the influence of different GelMA concentrations on SC viability. Figure 2C shows that the viability of SCs is much higher in 5% GelMA than in 10% GelMA, as evidenced by fewer dead cells in 5% GelMA (white arrow). Dead cells were counted to quantify cell viability further; results are shown in Figure 2D. The number of dead SCs in 10% GelMA was significantly larger than that in 5% GelMA.

**Figure 3:**
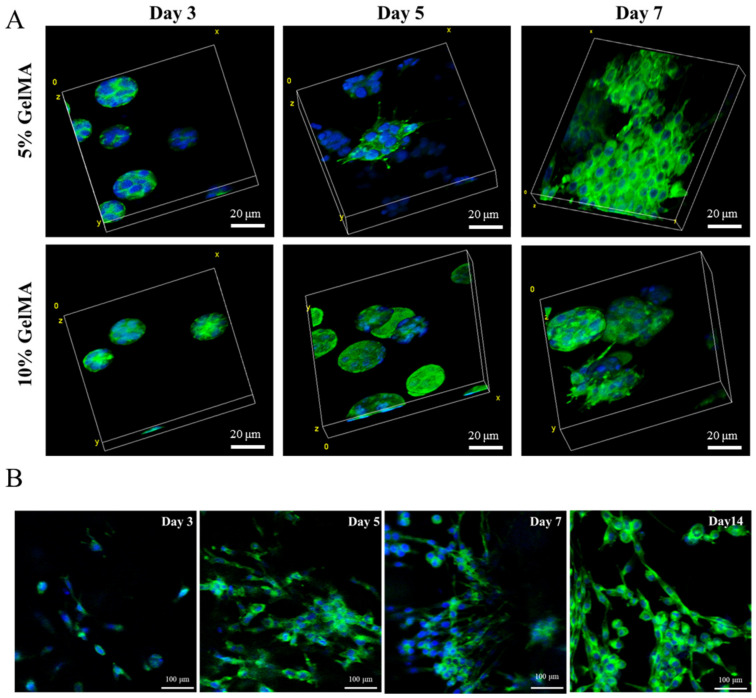
Cell morphology of SCs embedded 5% and 10% GelMA. (**A**) Confocal laser microscopy images of SCs stained with phalloidin (green) and cell nuclei (blue) after culturing 3, 5, and 7 days. (**B**) Immunofluorescent staining of SCs embedded in 5% GelMA after 3, 5, 7, and 14 days of culture.

Phalloidin/DAPI staining was used to investigate the influence of GelMA concentration on SCs morphology. The morphology of SCs in 5% GelMA had a similar round shape at day 3 as that in 10% GelMA but demonstrated a much more extensive morphology at day 5 and day 7 (Figure 3A). Similar results were observed in the Live/Dead assay (Figure 2C), where SCs had a significantly more extensive morphology in 5% GelMA than 10% GelMA. SCs were further cultured in 5% GelMA until day 14 to study the influence of long-time culture on cell extension. Figure 3B showed the SCs morphological extending process in 5% GelMA from day 3 to day 14, suggesting that SCs morphology can be maintained for at least two weeks.

**Figure 4:**
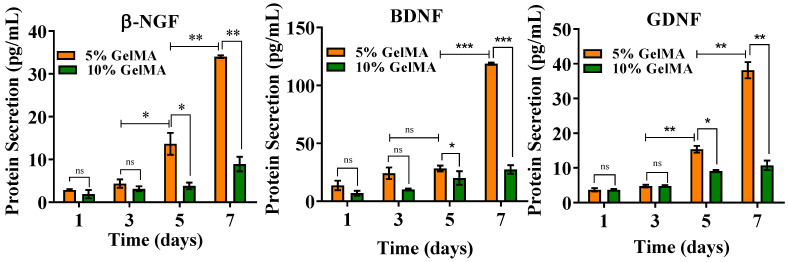
Neurotrophic factors secretion of SCs encapsulated in 5% and 10% GelMA. * *p* < 0.05, ** *p* < 0.01, *** *p* < 0.001.

To analyze the difference of neurotrophic factor secretions between 5% and 10% GelMA, we quantified the secretions of NGF, BDNF, and GDNF using ELISA assay. The supernatant of 5% and 10% GelMA samples were assessed using ELISA assay to study the effect of GelMA concentration on the secretion of neurotrophic factors from SCs. All three neurotrophic factors (NGF, BDNF, and GDNF) demonstrated higher secretion from day 1 to day 7 for all groups (Figure 4). Although the secretion of neurotrophic factors from SCs was significantly different in 5% and 10% GelMA at all time points, it was not apparent at the beginning of the cell culture. However, after day 5 and 7, the secreted amount of NGF, BDNF, and GDNF from SCs in 5% GelMA increased much faster than that in 10% GelMA. Especially on day 7, all three neurotrophic factors had a significantly higher secretion in 5% GelMA. The induction of nerve cell proliferation, adhesion and migration through neurotrophic factors have been well studied [49,50]. The neurotrophic factors secreted by SCs may also promote intercellular bridging and support neuronal survival [51].

Based on the SCs proliferation, viability, and axon extension and the secretion of neurotrophic factors, 5% GelMA was more suitable for SCs culture than 10% GelMA. Therefore, 5% GelMA was selected for the co-culture system to investigate the influence of SCs on PC-12 proliferation and differentiation.

### 2.3. PC-12 Cells Proliferation and Differentiation with SCs Co-Culture

**Figure 5:**
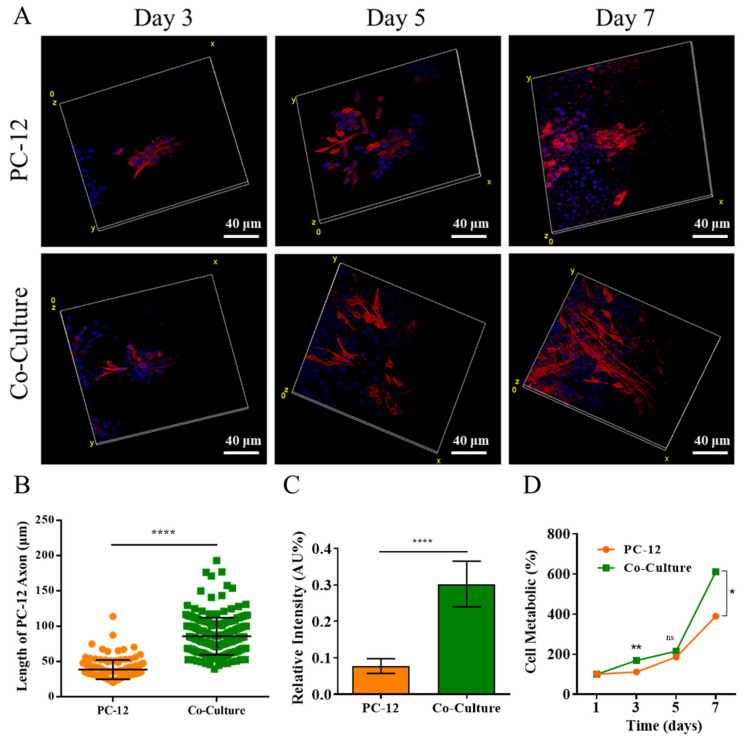
PC-12 cells proliferation and immunofluorescent staining in a co-culture system. (**A**) Confocal laser microscopy images of PC-12 cells stained with βIII tubulin (red) and cell nuclei (blue) after co-culture with SCs 3, 5, and 7 days. (**B**) Statistical axons of PC-12 cells. (**C**) Statistical immunofluorescent area of βIII tubulin. (**D**) Cck-8 result of PC-12 proliferation. * *p* < 0.05, **** *p* < 0.0001.

A co-culture system was used to study the effect of SCs on PC-12 axon extension and proliferation, where SCs were encapsulated in the 5% GelMA, and PC-12 cells were cultured on the GelMA surface. PC-12 cells were stained with βIII tubulin to investigate the axonal extension in the co-culture, as shown in Figure 5A. PC-12 cells had similar axonal extension at day 3 in co-culture compared with the control group, while the difference was more evident at day 5 and day 7, where PC-12 cells in co-culture had significantly longer axonal extensions than the control group. This observation was further supported by quantifying axon length for PC-12 cells at day 7 (Figure 5B), which may be caused by neurotrophic factors being secreted by SCs [13,14]. Indeed, the enhanced differentiation of neural stem cells has been observed in SC-neuron co-cultures [52,53]. Furthermore, the fluorescence intensity of βIII tubulin for PC-12 cells in co-culture was also determined to quantify the protein expression (Figure 5C), suggesting that PC-12 cells in co-culture had significantly higher βIII tubulin expression than the control group. In addition, beneficial neuronal effects from SCs in 3D co-cultures also have been reported previously [54,55].

The cellular proliferation of PC-12 cell was analyzed with CCK-8 assay. As shown in Figure 5D, PC-12 cells grow much faster in the co-culture system compared to the PC-12 only group (control group) at all time points. This difference was most evident on day 7, suggesting that SCs could promote the proliferation of PC-12 cells. Therefore, the co-culture revealed that the existence of SCs enhanced the axon extension of PC12 cells and promoted proliferation.

However, most studies used direct co-culture systems to mix SCs and neurons, allowing free cell-cell interactions through various communication pathways, such as transmembrane proteins and paracrine and chemical synapses, while causing the difficulties to investigate the mechanism of cell behavior change deeply. However, in this co-culture system, PC-12 cells were kept apart from SCs, which could be a better way to study the molecular mechanism of SC secretions on neuron behaviors.

### 2.4. Neurotrophic Factors Expression

**Figure 6:**
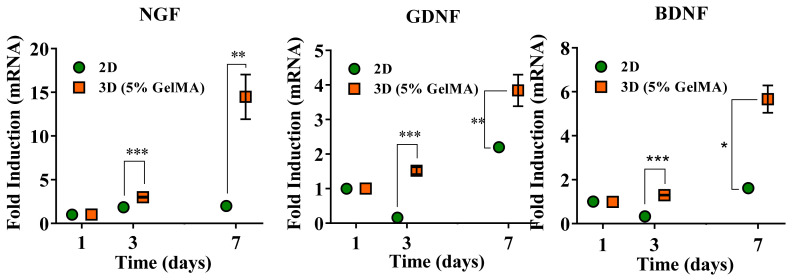
qPCR result of neurotrophic factors expression in 2D and 3D models. * *p* < 0.05, ** < 0.01, *** *p* < 0.001.

To further investigate the molecular mechanism of how SCs stimulate PC-12 behaviors, the mRNA level of NGF, BDNF, and GDNF expression from SCs in 2D and 5% GelMA were analyzed by qPCR to investigate the advantages of SCs 3D culture. As shown in Figure 6, the gene expression of neurotrophic factors for 2D cell culture did not change much from day 1 to day 7. However, the gene expression for SCs in 5% GelMA significantly increased with time and had a much higher expression at day 3 and day 7 when compared with the 2D culture. A similar result was found in another study, where SCs cultured in the gelatin-sodium alginate hydrogel expressed higher mRNA levels of NGF, BDNF, GDNF, and PDGF than with the 2D culture [29]. In general, compared to 2D culture, 3D-culture conditions make it easier for cell–cell interaction with an extracellular matrix in scaffolds, creating a cell-friendly environment and further providing a long-term state of cell–ECM interactions that may better simulate natural organizational conditions to provide appropriate physical and chemical cues and enhance various cellular metabolic activities during culture [44,56].

Therefore, the results above suggested that 5% GelMA was much better for SCs differentiation, proliferation, cell survival, and neurotrophic factors secretion than 10% GelMA. Furthermore, 5% GelMA played a positive role in the proliferation and differentiation of neural-like cells in the co-culture system, which indicated that the co-culture of SCs and PC-12 cells in 5% GelMA could be a promising method to promote the regeneration of the peripheral nerve.

## 3. Conclusions

This study performed co-cultures of SCs and PC-12 cells in the 5% GelMA scaffold since 5% GelMA could better support SCs proliferation, viability, and myelination and a higher neurotrophic factors secretion from SCs. These results suggest that SCs dynamically respond to GelMA stiffness, with changes in different DMS (30% and 50%). Furthermore, PC-12 cells demonstrated a higher cell proliferation rate and axon extension in co-culture, indicating that SCs could stimulate PC-12 growth and axon extension, which could be caused by the secretion of neurotrophic factors from SCs. In addition, the mRNA level of neurotrophic factors expressed in 5% GelMA culture was significantly upregulated compared with 2D culture, suggesting that this co-culture system could be a promising platform to investigate the mechanism of nerve regeneration. Further studies will assess the 5% GelMA potential to enable tissue cultures, such as primary tissue explants, to form functional neuronal networks to better mimic cell behavior in vivo.

## 4. Materials and Methods

### 4.1. Materials

Gelatin obtained from porcine skin tissue (250 bloom, Type B), and methacrylic anhydride (MA) was purchased from Aladdin Industrial (Shanghai, China). PDMS was purchased from Sylgard (Midland, MI, USA). The photoinitiator, lithium phenyl-2,4,6-trimenthylbenzoyphosphinate (LAP), was purchased from Engineering For Life (Suzhou, China). SCs (Schwann cell, RSC96) and PC-12 were purchased from the Cell Bank of the Chinese Academy of Sciences (Shanghai, China). Dulbecco’s Modified Eagle Medium (DMEM) and Roswell Park Memorial Institute medium (RPMI-1640) were obtained from Gibco (Big Cabin, OK, USA). Cell Counting Kit-8 (CCK-8) was purchased from Beyotime (Shanghai, China). The Live/Dead Viability Assay Kit, 4′,6-diamidino-2-phenylindole (DAPI), and phalloidin-FITC were bought from Solarbio (China). All the primers were synthesized by Sangon Biotech (Shanghai, China). The ELISA kit for β-NGF was obtained from R&D (Minneapolis, MN, USA); the other two ELISA kits for BDNF and GDNF were purchased from Abcam (Cambridge, UK).

### 4.2. Synthesis of GelMA

GelMA was synthesized by adapting a previously published method [33,34,57]. Briefly, gelatin was dissolved in a buffer solution containing Na_2_CO_3_ and NaHCO_3_ at 50 °C under continuous stirring for 3 h until fully dissolved. Next, various volumes (0.025/1 and 0.05/1) of MA were added drop-wise into the gelatin solution to synthesize GelMA with various degrees of methacryloyl substitution (DMS, 30% and 50%, respectively). After reacting in the dark for 3 h, samples were diluted with 5-fold deionized water and dialyzed for three days at room temperature with water changes three times per day. Finally, GelMA solutions were frozen at −80 °C, lyophilized for three days, and stored at −20 °C for further use.

### 4.3. Scanning Electron Microscope

A scanning electron microscope (SEM, TM-100, Hitachi, Tokyo, Japan) was used to observe the pore structures of GelMA hydrogels with different DMS and concentrations. To that end, the GelMA hydrogel samples (n = 3) were prepared as described above and freeze-dried overnight in a vacuum machine (DYYB-10, Shanghai Deyangyibang Instruments co., Ltd., Shanghai, China) to remove the aqueous phase. Before observation, dried samples were sputter coated with gold for 1 min using PELCO easiGlow™ Glow Discharge (Ted Pella Inc., Redding, CA, USA). The quantification of porosity and pore size was performed using NIH ImageJ.

### 4.4. Mechanical Characterization

The mechanical properties of GelMA hydrogels with different DMS and concentrations were characterized by unconfined compression. The GelMA hydrogel samples were crosslinked as mentioned above, and all mechanical tests were carried out by a dynamic mechanical analysis instrument (ElectroForce, TA Instruments, New Castle, DE, USA). For unconfined compression, GelMA hydrogel cylinders (*n* = 4, 2 mm thick, 12 mm diameter) were placed between two parallel plates and compressed at a displacement rete of 1 mm/min. Each test was run in triplicate at least. The compressive moduli were calculated as the slope of the linear region in the 0–10% strain range of the stress–strain curves.

### 4.5. Swelling Test

GelMA discs (*n* = 5) for 5% and 10% compositions were immersed in fully supplemented media and incubated at 37 °C and 5% CO_2_ for 5 d. The diameters were measured at 0, 0.5, 1.5, 4, 6, 24, 48, 72, and 120 h using a digital micrometer (Deli, Ningbo, China). The measurements were normalized to the diameter at 0 h to provide percent swelling values.

### 4.6. Cell Culture

SCs were cultured in DMEM (Gibco, Big Cabin, OK, USA) containing 10% fetal bovine serum (Excell Bio, Shanghai, China) and 1% penicillin/streptomycin (Big Cabin, OK, USA). In addition, PC-12 cells were cultured in RPMI-1640 supplemented with 10% FBS and 1% P/S. Cells were incubated in a humidified incubator (Thermo Scientific, Waltham, MA, USA) at 37 °C and 5% CO_2_. Cells used for experiments were in the log phase of growth and within passages P1 to P10.

### 4.7. SCs Encapsulation in GelMA

Two concentrations (5% and 10%) of GelMA precursor solution were prepared by dissolving in a fully supplemented cell culture medium carrying 0.5% (*w*/*v*) with LAP as a photoinitiator. SCs were trypsinized by 0.25% Trypsin-EDTA (Gibco, Big Cabin, OK, USA) when the cell reached 70–80% confluency and were resuspended in GelMA solution with a final cell density of 1 × 10^6^ cells/mL. The cell suspension was immediately added into polylactic acid (PLGA) molds (8 mm inner diameter, 2 mm thickness) and crosslinked with ultraviolet light (405 nm, 10 W/cm^2^) at a distance of 1 cm for 1 min, resulting in a cylinder GelMA encapsulated with SCs. These irradiation parameters have been optimized and shown to maximize the crosslinking of monomers chains. All samples of cell-laden GelMA were promptly transferred into 24-well tissue culture plates and incubated for seven days, and media was changed every other day.

### 4.8. Co-Culture of PC-12 Cells and SCs on GelMA

**Figure 7:**
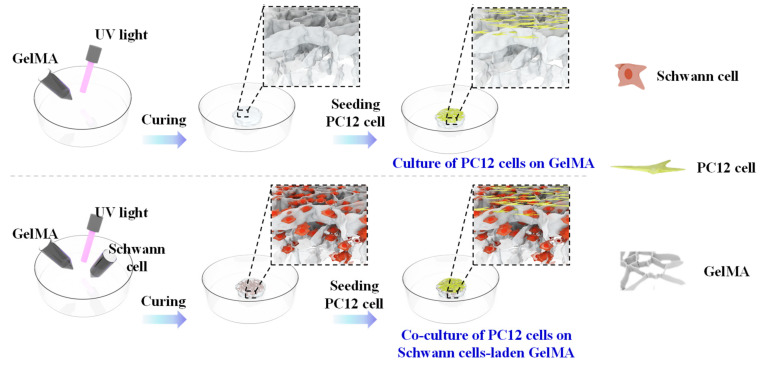
The schematic diagram of co-culture system.

The co-culture model was performed by seeding PC-12 cells on the SCs encapsulated GelMA surface (Figure 7). After the SCs encapsulated in GelMA were transferred to tissue culture plates, PC-12 cell suspension (2 × 10^5^ cells/mL) was added to the well and dropped freely on the GelMA surface. PC-12 cells seeded on the GelMA without SCs encapsulation were also prepared as a control group. After 24 h, samples were transferred to a new tissue culture plate to remove the PC-12 cells attached to the bottom of the plate.

### 4.9. Cellular Proliferation Analysis

CCK-8 assays (Beyotime, Shanghai, China) were carried out on days 1, 3, 5, and 7 to investigate the proliferation of the SCs with different concentrations of GelMA. At each time point, cell-laden samples (*n* > 3) were transferred into a new tissue culture plate to avoid the influence of cells growing on the bottom of the plate that dropped out from GelMA. CCK-8 assay was performed according to the manufacturer’s protocol, where 10 *v*/*v*% of CCK-8 working solution was prepared with fully supplemented media and incubated with samples for 2 h. The CCK-8 solution was then transferred to a 96-well plate for fluorescence reading at 450 nm. The cellular proliferation rate of each time point was evaluated by normalizing to the day 1 absorbance.

The cellular proliferation for the co-culture system was also evaluated using the same protocol mentioned above. PC-12 cells seeded on the surface of GelMA alone were used as the negative control to evaluate the PC-12 cell proliferation in co-culture—SCs encapsulated in GelMA alone were used as blanks. The PC-12 cell proliferation under the influence of SCs was obtained using the fluorescence intensity of the co-culture system to subtract the fluorescence intensity of SCs in GelMA alone at each time point.

### 4.10. Live-Dead Assay

A fluorescence live/dead assay (Calcein-AM/propidium iodide, Solarbio, Beijing, China) was performed based on the manufactory’s protocol to assess the viability of embedded SCs in 5% and 10% GelMA. First, samples were rinsed with 1× assay buffer from the kit three times and then incubated with 500 μL working solution (2 μM Calcein-AM and 4.5 μM PI) for 15 min in the incubator. Next, the stained samples were washed twice by 1× assay buffer before being imaged using an inverted phase-contrast fluorescence microscope (Leica, Wetzlar, Germany). Finally, dead cells were quantified using ImageJ (Version 1.53k, NIH, Bethesda, MD, USA) using obtained images (*n* > 3).

### 4.11. Immunofluorescent Assay

The immunofluorescence staining was used to evaluate the protein expression of SCs and PC-12 cells. Samples were fixed in 4% paraformaldehyde (PFA, Solarbio, Beijing, China) for 2 h, washed with PBS three times, permeabilized with 0.5% Triton X-100 (Solarbio, Beijing China) for 15 min, and blocked with blocking buffer (3% BSA) for 2 h at room temperature. Primary antibodies, S100β (Abcam Cambridge, UK, 1:100) and βIII tubulin (Abcam, Cambridge, UK, 1:200), were used for SCs and PC-12. After incubation at 4 °C overnight, three samples were washed with PBS and stained with secondary antibodies, goat anti-rabbit IgG (Abcam, Cambridge, UK, 1:500) and donkey anti-mouse IgG (Abcam, Cambridge, UK, 1:500) for 1 h at room temperature. In addition, cell nuclei were stained with DAPI (Solarbio, Beijing, China), and samples were washed with PBS three times before imaging by an inverted phase-contrast fluorescence microscope (Leica, Wetzlar, Germany). The axon extension and fluorescence intensity of PC-12 cells were evaluated using the images of βIII tubulin staining in combination with ImageJ.

Phalloidin staining was used for SCs encapsulated in GelMA on days 3, 5, 7, and 14 to analyze the cellular morphology. First, samples were fixed with 4% PFA and permeabilized with 0.5% Triton X-100 for 20 min at room temperature. Next, phalloidin (Solarbio, Beijing, China, 1:200) solution was applied for further incubation in the dark for 2 h, followed by DAPI staining for nuclei. Finally, the stained samples were washed with PBS three times and imaged using confocal microscopy (Nexcope, Ningbo, China).

### 4.12. Real-Time PCR

The RNAs of SCs cultured in 2D and 5% GelMA were harvested using TRIzol (Life, Waltham, MA, USA), further insolated using chloroform (Guoyao, Shanghai, China), and washed with isopropanol (Guoyao, Shanghai, China) once and 75% ethanol (Guoyao, Shanghai, China) twice. The extracted RNA was finally dissolved in RNase-free water, and the concentration was measured by Nanodrop (Thermo Scientific, Waltham, MA, USA). Complementary DNA (cDNA) was synthesized using the FastKing gDNA Dispelling RT SuperMix kit (TIANGEN, Beijing, China). Relative gene expression analysis was evaluated using real-time PCR (Roche, Basel, Switzerland). Power SYBR Green PCR Master Mix (Life, Waltham, MA, USA) was mixed with 50 ng cDNA and specific primers (Table 1, Sangon Biotech, Shanghai, China) in a total volume of 10 μL.

### 4.13. ELISA Assay for the Secretion of Neurotrophic Factors

The secretion of β-NGF (R&D, Minneapolis, MN, USA), BDNF (Abcam, Cambridge, UK), and GDNF (Abcam, Cambridge, UK) for SCs in GelMA were measured using an ELISA assay based on the manufacturer’s protocol. The supernatant of SCs laden GelMA (5% and 10%) at day 1, 3, 5, and 7 was collected and analyzed by a microplate reader (Molecular Devices, SpectraMax iD3, San Francisco, CA, USA).

### 4.14. Statistical Analysis

Unless otherwise stated, all results were analyzed using GraphPad 6.02. All the experiments were performed in triplicate. Data are represented as means ± standard deviations. One-way ANOVA analysis of variance was used for all data. A *p*-value < 0.05 was considered to be a statistically significant difference.

## Figures and Tables

**Table 1:** Primer sequence.

Primer Name	Sequence (F) 5′–3′	Sequence (R) 5′–3′
ACTIN	CCGCGAGTACAACCTTCTTG	CAGTTGGTGACAATGCCGTG
Ki67	CGCAGGAAGACTCGCAGTTT	CTGAATCTGCTAATGTCGCCAA
NGF	GGACGCAGCTTTCTATCCTGG	CCCTCTGGGACATTGCTATCTG
BDNF	TCATACTTCGGTTGCATGAAGG	AGACCTCTCGAACCTGCCC
GDNF	CTGACTTGGGTTTGGGCTAC	CCTGGCCTACCTTGTCACTT

## Data Availability

The original contributions presented in the study are included in the article, further inquiries can be directed to the corresponding authors.
